# Synaptic Activity Regulates Mitochondrial Iron Metabolism to Enhance Neuronal Bioenergetics

**DOI:** 10.3390/ijms24020922

**Published:** 2023-01-04

**Authors:** Paula Tena-Morraja, Guillem Riqué-Pujol, Claudia Müller-Sánchez, Manuel Reina, Ofelia M. Martínez-Estrada, Francesc X. Soriano

**Affiliations:** 1 Celltec-UB, Departament de Biologia Cellular, Fisiologia i Immunologia, Universitat de Barcelona (UB), 08028 Barcelona, Spain; 2 Institut de Neurociències (UBNeuro), Universitat de Barcelona (UB), 08035 Barcelona, Spain; 3 Institut de Biomedicina (IBUB), Universitat de Barcelona (UB), 08028 Barcelona, Spain

**Keywords:** iron, mitochondria, bioenergetics, neuron, oxygen consumption, synaptic activity, transcription

## Abstract

Synaptic activity is the main energy-consuming process in the central nervous system. We are beginning to understand how energy is supplied and used during synaptic activity by neurons. However, the long-term metabolic adaptations associated with a previous episode of synaptic activity are not well understood. Herein, we show that an episode of synaptic activity increases mitochondrial bioenergetics beyond the duration of the synaptic activity by transcriptionally inducing the expression of iron metabolism genes with the consequent enhancement of cellular and mitochondrial iron uptake. Iron is a necessary component of the electron transport chain complexes, and its chelation or knockdown of mitochondrial iron transporter Mfrn1 blocks the activity-mediated bioenergetics boost. We found that Mfrn1 expression is regulated by the well-known regulator of synaptic plasticity CREB, suggesting the coordinated expression of synaptic plasticity programs with those required to meet the associated increase in energetic demands.

## 1. Introduction

The brain is the most energy-demanding organ in the body. Accounting for 2% of the body mass, the brain consumes 20% of the oxygen used by the resting body. Around 75% of the energy expenditure in the brain is used in processes related to synaptic transmission, such as reestablishment of ion balance or neurotransmitter recycling [1]. As a consequence of synaptic activity, there are structural and functional adaptations in which synapses are potentiated, resulting in an increase in the strength of these synapses, a form of synaptic plasticity that is involved in learning and memory [2]. This synaptic strengthening also increases energy expenditure and, therefore, requires an increased ATP supply [1]. Most of the ATP generated in the neuron is produced by mitochondrial oxidative phosphorylation (OXPHOS) [1]. Therefore, neurons with persistent synaptic activity must experience long-lasting bioenergetics remodelling to support increased activity-related energetic demand. Understanding the mechanism by which this remodelling takes place has important implications for enhancing neuronal metabolism in conditions, such as aging, in which bioenergetics capabilities decline [3,4].

Iron is an essential metal used in several oxidation/reduction reactions involved among others in DNA biosynthesis and repair, oxygen transfer and energy production [5,6]. Iron is taken up into the cell through receptors and transporters [6]. Intracellular iron is stored by cytosolic ferritin or targeted to mitochondria, which is the major cellular site of iron utilization [7]. Iron enters the mitochondrial matrix through the mitochondrial iron transporters mitoferrins (Mfrn1 and Mfrn2) [8]. Within the mitochondria, iron is used for the synthesis of heme and iron–sulfur clusters (ISC) proteins or stored in mitochondrial ferritin (FTMT) [7]. Iron in its ferrous state is exported out of the cell via ferroportin (FPN) [9,10,11].

Iron-containing heme and ISC proteins are essential components of electron transport chain complexes and some enzymes of the tricarboxylic cycle (TCA) [12,13]. During OXPHOS, electrons are transferred from electron donors, mainly produced in the TCA, to mitochondrial complexes within the inner mitochondrial membrane (IMM) that constitute the electron transport chain. The transfer of electrons from one mitochondrial complex to another is coupled with the transfer of protons to the intermembrane space and across the IMM to generate an electrochemical proton gradient that drives the synthesis of ATP. Heme shortage compromises mitochondrial respiration, as heme groups are required for anchorage and activity of respiratory complexes [12]. Therefore, alterations in mitochondrial iron content disturb mitochondrial ATP production [14,15].

Herein, we show that an episode of synaptic activity enhances mitochondrial metabolism by regulating neuronal iron metabolism at the transcriptional level, as shown by the impairment of activity-dependent bioenergetics when neurons are treated with an iron-chelating molecule or by knocking down the expression of the mitochondria iron uptake protein Mfrn1. Mfrn1 is regulated by CREB, and consequently, the expression of dominant negative A-CREB also blocks the activity-mediated bioenergetic boost, suggesting the coordinated expression of plasticity and bioenergetics genes.

## 2. Results

### 2.1. Synaptic Activity Enhances Mitochondrial Bioenergetics

**Figure 1:**
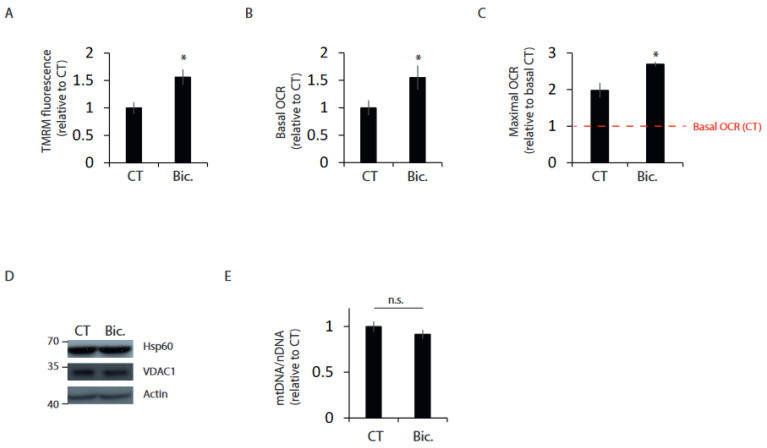
Synaptic activity enhances mitochondrial bioenergetics. Primary cortical neurons were stimulated with bicuculline plus 4-AP (labeled Bic in this and subsequent figures) for 24 h or left unstimulated (CT), 30 min after washing out to block burst firing: (**A**) mitochondrial membrane potential (MMP) was determined by measuring TMRM fluorescence (n = 4 independent experiments). (**B**) Basal and (**C**) maximal oxygen consumption rate (OCR) was analyzed (n = 3 independent experiments). (**D**) Representative Western blots of protein samples of control and stimulated neurons (n = 3 independent experiments). (**E**) Mitochondrial and nuclear DNA was analyzed by qPCR (n = 4 independent experiments). * *p* < 0.05, two-tailed Student’s *t*-test. n.s. = non-significant.

Synaptic function accounts for most of the energetic expenditure of neurons [1]. Therefore, we studied whether there are modifications to mitochondrial energetics in neurons that have experienced an episode of synaptic activity. We used an established method of network disinhibition to enhance synaptic activity in cultures of primary cortical neurons by applying the GABAA receptor antagonist bicuculline (Bic) and the K^+^ channel antagonist 4-aminopyridine (4AP) [16]. After 24 h of synaptic stimulation, neurons were washed for 30 min to block burst firing [17]. Neurons that had been previously stimulated showed increased mitochondrial membrane potential and higher basal and maximal oxygen consumption (OCR) (Figure 1A–C and Figure S1A). Synaptic activity induces the expression of the master regulator of mitochondrial biogenesis PGC1A [18], and therefore, to study the possibility that higher OCR was due to increased mitochondrial mass, mitochondrial mass was analyzed in resting and 24 h stimulated neurons. We used two strategies, the Western blot of two commonly used mitochondrial mass markers, VDAC1 and HSP60, and the quantitative determination of mtDNA. Both techniques showed that, 24 h after synaptic stimulation, there was no change in mitochondrial mass (Figure 1D,E). In agreement with other reports [17,19,20], stimulation did not affect neuronal viability (Figure S2), ruling out the possibility of a selection of more respiratory neurons.

Hence, increased mitochondrial bioenergetics in neurons that have experienced an episode of synaptic activity is not caused by increased mitochondrial mass but by some type of mitochondrial adaptation.

### 2.2. Increased Intracellular Iron Levels in Active Neurons Participate in Bioenergetics Boost

**Figure 2:**
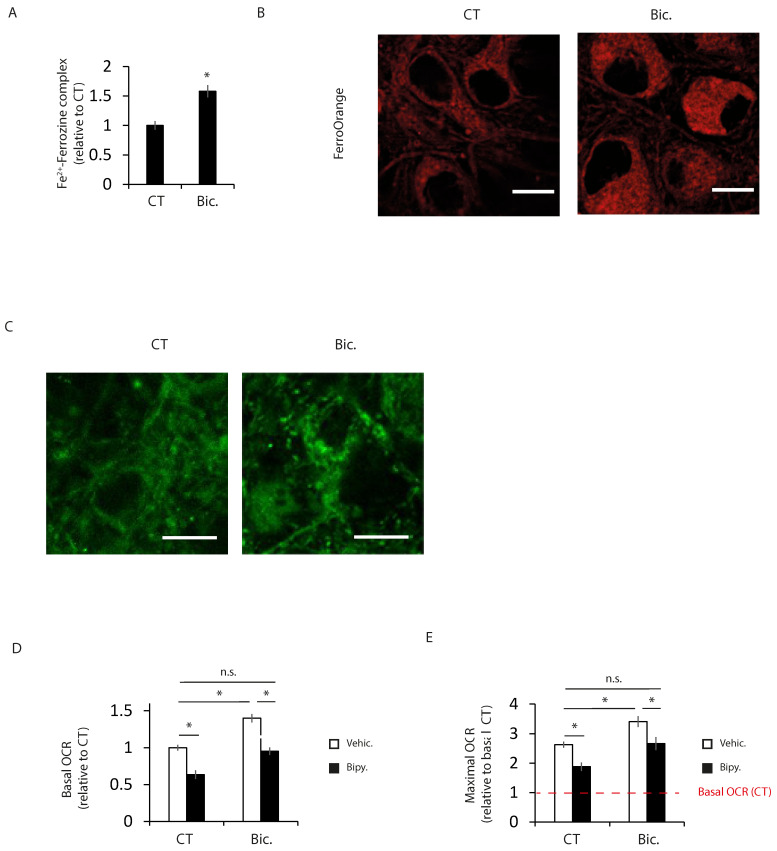
Increased intracellular iron levels in active neurons participate in bioenergetics boost. (**A**) Ferrous iron content in control unstimulated and 24 h Bic-stimulated neurons was analyzed using the ferrozine-based assay (n = 4 independent experiments). * *p* < 0.05, two-tailed Student’s *t*-test. (**B**) Representative images of FerroOrange staining in control and stimulated neurons (n = 3 independent experiments). Scale bar = 10 µm. (**C**) representative images of Mito-FerroGreen staining in control and stimulated neurons (n = 4 independent experiments). Scale bar = 10 µm. (**D**) Basal and (**E**) maximal oxygen consumption rate (OCR) was analyzed in neurons treated with the iron chelator 2,2′-bipyridyl (bipy, 100 µM) (n = 4 independent experiments). The red dashed line indicates basal OCR of resting neurons. * *p* < 0.05, one-way ANOVA, followed by Tukey’s post hoc test. n.s. = non-significant.

**Figure 3:**
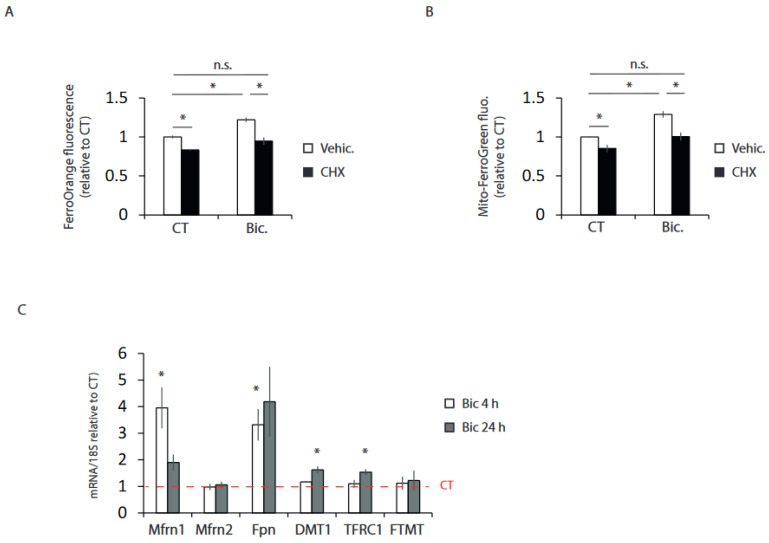
Synaptic activity induces the transcriptional expression of iron metabolism genes. (**A**) Cytoplasmic and (**B**) mitochondrial ferrous content was analyzed in control and 24 h stimulated neurons in absence or presence of the translation inhibitor cycloheximide (10 µM) as indicated (n = 3 and 4 independent experiments, respectively). (**C**) Neurons were stimulated for 4 or 24 h, and mRNA expression of the indicated genes was determined by qPCR (n = 4–8 independent experiments). The red dashed line indicates mRNA levels in resting neurons * *p* < 0.05, one-way ANOVA, followed by Tukey’s post hoc test except for Fpn and FTMT qPCR results, which used Kruskal–Wallis, followed by Dunn’s post hoc test. n.s. = non-significant.

Neuronal activity enhances iron uptake by the neuron [21,22,23]. Iron is an essential cofactor for important biochemical reactions, such as oxygen transport and energy metabolism. Hence, we analyzed intracellular iron using the ferrozine-based colorimetric assay and a fluorescent ferrous iron (Fe^2+^) sensor. Both techniques showed increased levels of intracellular iron in active neurons (Figure 2A,B and Figure 3A). Intracellular iron is mainly used in mitochondria, where it is used for the synthesis of iron–sulfur clusters (ISC) and the heme group, which act as cofactors of multiple enzymes, including TCA cycle enzymes and OXPHOS complexes. Using a mitochondria-targeted fluorescent sensor, we observed increased levels of iron in the mitochondria of stimulated neurons (Figure 2C and Figure 3B). Increased mitochondrial iron content in neurites was similar to that in soma (Figure S3). 

To investigate whether an activity-mediated increase in iron levels is important for enhancing mitochondrial bioenergetics, neurons were treated with the iron chelator 2,2′-bipyridyl (bipy). Bipy treatment impaired the synaptic activity-mediated increase in basal and maximal oxygen consumption (Figure 2D,E and Figure S1B,C).

All together, these results indicate that synaptic-activity-mediated iron uptake plays an important role in enhancing neuronal bioenergetics.

### 2.3. Synaptic Activity Induces the Transcriptional Expression of Iron Metabolism Genes

Through the generation of Ca^2+^ transients, synaptic activity is one of the most potent regulators of gene expression [24,25]. To examine the possibility that increased iron uptake induced by synaptic activity requires the synthesis of new proteins, neurons were treated with the translation inhibitor cycloheximide (CHX), and we observed that CHX treatment completely blocked the activity-mediated rise in intracellular and mitochondrial iron (Figure 3A,B). These data suggested that transcriptional changes might be associated with increased iron uptake in neurons that have experienced synaptic activity. Therefore, we analyzed the mRNA levels of different genes involved in iron metabolism. We observed strong induction of Mfrn1, which mediates mitochondrial iron import, and ferroportin (Fpn), which mediates cellular iron export (Figure 3C). A more modest, although significant, induction was observed in transferrin receptor (TFRC1) gene expression, which is necessary for receptor-mediated cellular iron uptake, and DMT1, which mediates iron transport across the endosomal membrane to the cytosol (Figure 3C).

These results indicate that synaptic activity regulates the expression of iron metabolism genes in a coordinated manner.

### 2.4. Mfrn1 Knockdown Impairs Mitochondrial Bioenergetics

**Figure 4:**
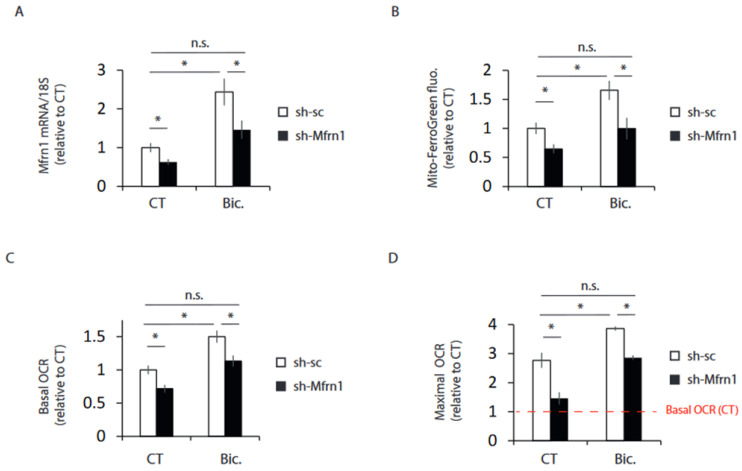
Mfrn1 knockdown impairs mitochondrial bioenergetics. Neurons transduced with AAV expressing shRNA targeting Mfrn1 (shMfrn1) or control non-targeting shRNA (shsc). (**A**) After 4 h stimulation with Bic, Mfrn1 mRNA expression was determined by qPCR (n = 6 independent experiments). (**B**) Mitochondrial ferrous content was analyzed in control, and 24 h stimulated neurons transduced with an AAV expressing a shRNA targeting Mfrn1 (shMfrn1) or a non-targeting shRNA (shsc) as indicated (n = 4 independent experiments). (**C**) Basal OCR and (**D**) maximal OCR was determined after 24 h stimulation with Bic (n = 5 independent experiments). The red dashed line indicates basal OCR of resting neurons. * *p* < 0.05, one-way ANOVA, followed by Tukey’s post hoc test. n.s. = non-significant.

Mitochondria is the major site of iron utilization, and it acts as a cofactor of different proteins involved in bioenergetics. Thus, we knocked down Mfrn1 using an AAV expressing a shRNA targeting Mfrn1 (AAV-shMfrn1) in order to study whether blockage of increased iron uptake induced by synaptic activity also impaired the enhancement of bioenergetics. As a functional consequence of the blockage of activity-dependent induction of Mfrn1 by AAV-shMfrn1 (Figure 4A), Mfrn1 KD neurons showed reduced mitochondrial iron levels (Figure 4B). Mfrn1 KD neurons showed diminished mitochondrial bioenergetics enhancement after stimulation (Figure 4C,D and Figure S1D,E), further supporting the view that an increase in iron metabolism is necessary to fully enhance mitochondrial bioenergetics in active neurons.

### 2.5. Mfrn1 Is Regulated by CREB

**Figure 5:**
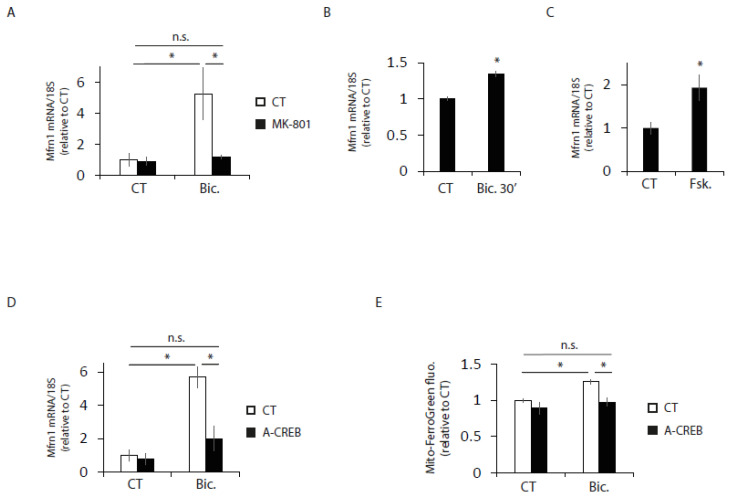
Mfrn1 is regulated by CREB. (**A**) Neurons were stimulated with Bic for 4 h with or without MK-801 (10 µM), and Mfrn1 mRNA expression was determined by qPCR (n = 5 independent experiments). * *p* < 0.05, one-way ANOVA, followed by Tukey’s post hoc test. (**B**) Neurons were stimulated for 30 min, and Mfrn1 mRNA expression was determined by qPCR (n = 4 independent experiments). * *p* < 0.05, two-tailed Student’s *t*-test. (**C**) Neurons were treated with forskolin (FSK, 10 µM) for 4 h, and Mfrn1 mRNA expression was determined by qPCR (n = 4 independent experiments). * *p* < 0.05, two-tailed Student’s *t*-test. (**D**) Neurons were transduced with AAV encoding the dominant negative A-CREB, and after 4 h stimulation with Bic Mfrn1, mRNA expression was determined by qPCR (n = 3 independent experiments). * *p* < 0.05, one-way ANOVA, followed by Tukey’s post hoc test. (**E**) Analysis of Mito-FerroGreen fluorescence intensity in CT and AAV-CREB transduced neurons left unstimulated or stimulated with Bic for 24 h. (n = 3 independent experiments). * *p* < 0.05, one-way ANOVA, followed by Tukey’s post hoc test. n.s. = non-significant.

Since blocking Mfrn1 induction is sufficient to impair the activity-dependent enhancement of mitochondrial respiratory capacity, we next aimed to identify the transcription factor that regulates Mfrn1 induction in active neurons. The expression pattern of Mfrn1 after stimulation resembles that of CREB-dependent activity-regulated genes [17,26,27]. CREB is one of the most studied transcription factors involved in synaptic activity-regulated transcriptional changes, which are involved in important neurobiological processes, such as memory, learning and neuronal survival [24,25,28]. CREB also regulate the expression of genes involved in the metabolism in neurons and peripheral tissues [17,29,30]. Synaptic activity causes CREB activation initiated by calcium flux through the NMDA receptor (NMDAR) [19]. In agreement with a possible role of CREB regulating the activity-mediated induction of Mfrn1, we first observed that the NMDAR antagonist MK-801 blocked activity dependent induction of Mfrn1 (Figure 5A). In addition, a small but significant induction of Mfrn1 mRNA was observed after just 30 min of stimulation (Figure 5B), consistent with the rapid CREB activation triggered by synaptic activity [16,31,32,33]. Therefore, we reasoned that CREB might regulate Mfrn1 in active neurons. We first treated neurons with the adenylyl cyclase activator and thus, CREB activator, forskolin. Forskolin treatment was sufficient to induce Mfrn1 (Figure 5C), suggesting that CREB regulates Mfrn1 expression.

To confirm that CREB is the transcription factor by which synaptic activity induces Mfrn1, we transduced neurons with AAV expressing A-CREB, which is CREB dominant negative [34], and observed that the activity-mediated induction of Mfrn1 expression was blocked (Figure 5D). Consistent with the role of CREB in regulating activity-dependent Mfrn1 expression, we observed that A-CREB expression abolished the activity-dependent enhancement of mitochondrial iron uptake (Figure 5E). Hence, these results identify Mfnr1 as a new CREB target gene in active neurons that contributes to enhancing mitochondrial bioenergetics.

## 3. Discussion

In this study, we show that the mitochondria of neurons that have experienced synaptic activity display enhanced bioenergetics that last beyond the period of activity. The bioenergetics enhancement requires increased mitochondrial iron uptake, which is essential in complexes of the OXPHOS system [12].

Processes related to synaptic activity, such as the restoration of ionic gradients or synaptic vesicle recycling, require a huge energetic effort on the part of the neuron [1]. There are different mechanisms for dealing with this energetic stress meanwhile the synaptic activity takes place. For instance, an activity-dependent increase in Ca^2+^ keeps motile mitochondria at presynaptic sites to produce energy where it is most needed [35,36,37]. However, the activity-dependent increase in Ca^2+^ is transient, and once cytoplasmic Ca^2+^ levels are stabilized, mitochondria can restart movement. In addition, it has been shown that synaptic activity activates the master sensor of energy stress AMPK, which regulates glycolysis and mitochondrial respiration to increase energy production during synaptic activity [38,39]. Moreover, synaptic activity causes the translocation of the glucose transporter Glut4 to cell membrane to increase glucose levels [40], but the membrane translocation/endocytosis rates are likely to be restored towards intracellular retention once the translocation stimulus ceases, as in peripheral tissues [41]. In as short as an hour after stimulation, Rheb expression is induced and translocated to the mitochondrial matrix, where it enhances the activity of mitochondrial pyruvate dehydrogenase [42]. However, the long-lasting effects of synaptic activity on neuronal metabolism have been poorly studied. The surface of mitochondria crista strongly correlates with bioenergetics capacity. Indeed, the remodelling of crista architecture takes place as a response to increased energy demand [43,44]. An ultrastructural study of mitochondria of fast-spiking parvalbumin-positive basket cells and slow firing type-1 cannabinoid-positive basket cells showed higher crista density in the fast-spiking neurons, suggesting the activity-dependent ultrastructural remodeling of mitochondria [45]. Proteomics analysis showed differences in proteins of synaptic and non-synaptic mitochondria [46], although care must be taken in the interpretation of these findings, since in the analyzed samples, non-synaptic mitochondria were not limited to neuronal mitochondria but also included glia and other non-neuronal brain cells mitochondria. In this study, we have shown a novel mechanism by which an episode of synaptic activity enhances mitochondrial metabolism, although activity has ceased, based on increasing mitochondrial iron levels, which is a necessary component of the electron transport complexes in the mitochondria. 

Brain development and cognitive function requires a high amount of energy, and iron is necessary for proper mitochondrial function. Consequently, iron deficiency during late fetal and early postnatal life causes learning and memory impairments [47,48,49,50]. Moreover, iron is an essential metal that functions as a cofactor of different enzymes involved in neurotransmitter synthesis, and in oligodendrocytes, it is required for myelination [51,52]. Therefore, iron is necessary for proper brain function. However, an excess of iron is associated with aging and neurodegenerative disorders [6,53]. Iron can participate in the production of reactive oxygen species (ROS) and iron-dependent lipid peroxidation via the Fenton reaction, causing a regulated type of cell death known as ferroptosis [54], in which one of the hallmarks is the accumulation of iron in cells [55]. Ferroptosis and iron accumulation have been reported in neurodegenerative disorders, such as Alzheimer’s disease, Parkinson’s disease, Friedreich’s ataxia, among others [6,53]. Therefore, cellular iron levels need to be tightly regulated. Herein, we show that synaptic activity induces, at a transcriptional level, the expression of genes involved in intracellular iron import (TRFC1), transport from endosome to cytosol (DMT1) and mitochondrial iron uptake (Mfrn1), but synaptic activity also strongly induces the expression of Fpn that mediates cellular iron efflux. This apparent contradiction (simultaneous induction of iron uptake and efflux genes) might represent a defense mechanism against ferroptosis by achieving balanced intracellular iron levels. Indeed, loss of Fpn causes ferroptosis [56,57], and the enhancement of cellular iron export make cells more resistant to ferroptosis [58,59]. In addition to the upregulation of Fpn, active neurons also induce, at the transcriptional level, the expression of the system xc- cysteine-glutamate antiporter and glutathione synthesis, which is the main ferroptosis-controlling pathway [60,61,62]. All this suggests that synaptic activity, while increasing intracellular iron levels, coordinately regulates a transcriptional program to protect against ferroptosis, as occurs with other regulated cell death subroutines [63,64,65,66]. However, additional studies are needed to confirm this.

By activating different transcription factors, synaptic activity is one of the strongest inducers of gene expression [24]. Our data indicate that CREB is the transcription factor responsible for the activity-dependent induction of Mfrn1 expression. In neurons, CREB has mainly been studied in relation to its role as a regulator of plasticity, cell survival, and neurite outgrowth [24,25,28,67]. All these processes require increasing energy demands. The role of CREB in regulating the expression of metabolic genes has been studied mainly in peripheral tissues [29], although in neurons, it also has been described a metabolic role of CREB by regulating the expression of Glut3 [17,30], the main glucose transporter in neurons, and indirectly regulating rate-limiting glycolysis genes [17]. Herein, we show that the CREB-dependent induction of Mfrn1 is needed to enhance mitochondrial bioenergetics, preparing neurons for dealing with the energetic stresses associated with synaptic activity. In accordance with our results, a very recent study showed the regulation of Mfrn1 expression by CREB in the hepatoma PLC cell line [68]. Thus, our and others results suggest that CREB regulates in a coordinated manner the expression of genes important for neuronal function and the expression of bioenergetic genes necessary to satisfy the high energetic requirements of these neuronal processes.

In conclusion, we have unveiled a mechanism by which synaptic activity enhances mitochondrial bioenergetics beyond the period of activity, which may be relevant to dealing with the increasing energetic demand associated with synaptic activity.

## 4. Methods

### 4.1. Cell Culture and Stimulation

Cortical neurons from E21 Sprague Dawley rats were cultured in NBA (Neurobasal A medium + B27, 1% FBS and 1 mM glutamine) as described previously [69]. Experiments were performed after 10–11 days in culture, during which cortical neurons develop a rich network of processes, express functional NMDA-type and AMPA/kainate-type glutamate receptors, and form synaptic contacts. Bursts of action potential firing were induced by treating neurons with 50 µM bicuculline (Merck, Sigma-Aldrich, Burlington, VT, USA), and burst frequency was enhanced by adding 250 µM 4-amino pyridine (Merck, Sigma-Aldrich, Burlington, VT, USA).

### 4.2. Transfection, Plasmids and Virus Generation

Prior to transfection, neurons were transferred from growth medium to a medium composed of 10% MEM (Invitrogen) and 90% salt–glucose–glycine (SGG) medium (SGG: 114 mM NaCl, 0.219% NaHCO_3_, 5.292 mM KCl, 1 mM MgCl_2_, 2 mM CaCl_2_, 10 mM HEPES, 1 mM glycine, 30 mM glucose, 1 mM glutamine, 0.5 mM sodium pyruvate, 0.1% phenol red; osmolarity 325 mosm/L). Neurons were transfected at DIV8 using Lipofectamine 2000 (Thermo Fisher, Invitrogen, Carlsbad, CA, USA). Transfection efficiency was approximately 5%, with almost all transfected cells beeing neurons. 

The vectors used to construct and package recombinant adeno-associated viruses (rAAVs), pAAV-sh-sc, were kindly provided by Hilma Bading [70]. rAAV for shRNA expression contains the U6 promoter for shRNA expression and a CMV/chicken beta-actin hybrid promoter driving hrGFP expression. rAAV-shMfnr1 was made by swapping the sh-sc sequence of rAAV-sh-sc for the following sequence of the rat gene into the BamHI and HindIII restriction sites: 5′-GCC TGA ACG TGA TGA TGA T-3′.

A-CREB [70] was amplified using the following primers: A-CREB-F: 5′- TTT*A GAT CT*G CCA CCA TGG ACT ACA AGG ACG-3′ and –R: 5′- TTT *TCC GGA* ATC TGA CTT GTG GCA GTA AAG GTC-3′. The amplified product contains sequences with BglII and BspEI restriction sites at the 5′ and 3′, respectively (italics). A BspEI restriction site was introduced before the P2A sequence in AAV-hSyn1-GCaMP6f-P2A-NLS-d-Tomato (Addgene plasmid #51085; http://n2t.net/addgene:51085 accessed on 30 December 2022; RRID:Addgene_51085; a gift from Jonathan Ting), GCaMP6f was removed by digestion with BamHI and BspEI, and amplified A-CREB was cloned into this linearized vector.

Neurons were infected with rAAV at DIV4. Infection efficiencies were determined at DIV 10-11 by analyzing GFP or d-Tomato fluorescence or conducting an immunocytochemical analysis and were observed to range from 70 to 85% of the viable neurons.

All newly generated constructs were confirmed by sequencing.

### 4.3. RNA Isolation, RT-PCR and qPCR

RNA was isolated using a PureLinkTM RNA mini kit (Thermo Fisher, Life Technologies, Carlsbad, CA, USA). For qPCR; cDNA was synthesized from RNA using the SuperScript^®^ III First-Strand Synthesis SuperMix (Thermo Fisher, Life Technologies, Carlsbad, USA), following the manufacturer’s instructions. qPCR was performed in a StepOne Real-Time PCR System (Thermo Fisher, Applied Biosystem, Carlsbad, USA) using GoTaq qPCR Master Mix (Promega, Madison, WI, USA) according to the manufacturer’s instructions. The primers used were:

Mfrn1 -F: 5′- gta tgg cca ccc tac tcc ac -3′, -R: 5′- gca atc gct gtt tca cca c -3′; Mfrn2 -F: 5′- gga tgt gtg gca acg tta ctt -3′, -R: 5′- tcc gct gct tga cca ctt -3′; FPN –F: 5′- gca aac tac ctg acc tca gca -3′, -R: 5′- act gca aag tgc cac atc c -3′; DMT1 –F: 5′- cgg cca gtg atg agt gag t -3′, -R: 5′- agc aga cga gaa gga cca ag -3′; TFRC1–F: 5′- ccc ttc tcg aga tgc aac at-3′, -R: 5′- tcc agc ctc acg agg agt at-3′; FTMT –F: 5′- gcc aga act ttc acc cag ac -3′, -R: 5′- gga tgc gta aag ctc cat gt-3′; 18S -F: 5′-GTG GAG CGA TTT GTC TGG TT-3′, -R: 5′-CAA GCT TAT GAC CCG CAC TT-3′. Expression of the gene of interest was normalized to that of 18S rRNA, a commonly used control. 

For analysis of mtDNA/nDNA total DNA was isolated with the QIAamp DNA Mini Kit (QIAGEN, Hilden, Germany). Real-time qPCR was performed with primers mt-COXI -F: 5′- GCT TCG TCC ACT GAT TCC CA -3′, -R: 5′- GCA AAG TGG GCT TTT GCT CA -3′ and nFabp1: -F: 5′- ATG GGC CAC GAT CTG TCT TC -3′, -R: 5′- GTG GCA AGA CCA GAG TGT CA -3′ to amplify mitochondrial and nuclear DNA, respectively.

### 4.4. Western Blotting and Antibodies

Total cell lysates were boiled at 100 °C for 5 min in 1.5× sample buffer (1.5 M Tris pH 6.8; 15% glycerol; 3% SDS; 7.5% β-mercaptoethanol; 0.0375% bromophenol blue). Gel electrophoresis was performed using 9% polyacrylamide gels. The gels were blotted onto PVDF membranes, which were then blocked for 1 h at room temperature with 5% (*w/v*) non-fat dried milk in PBS with 0.05% Tween 20. The membranes were then incubated overnight at 4 °C with the primary antibodies diluted in blocking solution as follows: VDAC1 (1:1000; Ab14734; Abcam, Cambridge, UK), HSP60 (1:2000; Ab190828; Abcam) and actin (1:1000, A4700, Merck, Sigma-Aldrich, Burlington, VT, USA). To visualize Western blots, HRP-based secondary antibodies were used, followed by chemiluminescent detection on Kodak X-Omat film.

### 4.5. Oxygen Consumption Assay

Extracellular oxygen consumption was measured using the MitoXpres Xtra Oxygen Consumption Assay kit (Agilent, Santa Clara, CA, USA) following the manufacturer’s instructions. Briefly, cells were grown in 96-well plates to confluence. Before the assay, the culture medium was replaced with fresh NBA plus MitoXpress Xtra reagent (diluted 1:10), and two drops of mineral oil were promptly added. Plates were read at 380 nm excitation and 650 nm emission, every minute for 3 h. Maximal OCR was assayed after the addition of CCCP (10 µM). For analysis, after an initial decrease in fluorescence due to O_2_ back diffusion through the body of the microplate reader [71] the fluorescence signal started to increase in a linear fashion for at least the next two hours. Fluorescence values were plotted against time, and the slope of the linear portion was determined. The values were normalized by total protein levels, quantified using a Pierce BCA Protein Assay Kit (Thermo Fisher, Carlsbad, CA, USA).

### 4.6. Analysis of Mitochondrial Membrane Potential 

To quantify MMP, cells were loaded for 30 min at 37 °C with tetramethylrhodamine methylester (TMRM; Merck, Sigma-Aldrich, Burlington, VT, USA) at a non-quenching concentration (6 nM) in phenol red-free HBSS with 10 mM HEPES and 5.5 mM glucose (Gibco, 14025-050; Life Technologies, Carlsbad, CA, USA). Single cells were monitored, TMRM was excited at 540 nm, and emission was measured using a 570 nm filter. Background values obtained after mitochondrial depolarization with CCCP (10 µM, the concentration that in titration experiments completely depolarized mitochondria) were subtracted from the TMRM fluorescence values. 

### 4.7. Measurement of Cellular and Mitochondrial Iron Levels

Iron content was measured using a colorimetric ferrozine-based assay. Cell lysate mixed with concentrated HCl (11.6 M) was heated at 95 °C for 20 min and then centrifuged at 12,000× *g* for 10 min. Ascorbate was added to the supernatant, and, after 2 min of incubation at room temperature, ferrozine and saturate ammonium acetate (NH4Ac) were sequentially added. The absorbance was measured at 570 nm. The values were normalized by total protein levels, quantified using a Pierce BCA Protein Assay Kit (Thermo Scientific, Carlsbad, CA, USA).

FerroOrange (Dojindo) and Mito-FerroGreen (Dojindo) were used to measure the amount of intracellular and mitochondrial ferrous iron (Fe^2+^) content, respectively, according to the manufacturer’s instructions. Briefly, neurons were incubated for 30 min at 37 °C with FerroOrange at 1 μmol/L or Mito-FerroGreen at 5 μmol/L in HBSS. The fluorescence signals (Ex/Em = 543 nm/580 nm for FerroOrange and 488/550 nm for Mito-FerroGreen) were measured on a Leica confocal laser scanning microscope (Life Imaging Services, Basel, Switzerland). Before the observation of Mito-FerroGreen, neurons were washed three times with HBSS. ROIs of the same surface were drawn in the soma, and fluorescence intensity was analyzed using ImageJ [72]. Fluorescence intensity in resting and neurons that have experienced an episode of synaptic activity was within the range obtained when neurons were treated with ammonium iron (II) sulfate (100 µM) and treated with the iron chelator bipyridyl (100 µM).

### 4.8. Statistical Analysis

Normal distribution of samples was tested using a Shapiro–Wilk test. For normal distributed data, statistical testing involved two-tailed Student *t*-tests for comparisons of two groups or for multiple comparisons within data sets, we used one-way ANOVA, followed by Tukey’s post-hoc test. For nonparametric multiple comparisons test, we used Kruskal–Wallis, followed by Dunn’s posthoc test. All data are presented as mean ± S.E.M. of at least three independent experiments (n).

## Data Availability

This study includes no data deposited in external repositories. The data presented in this study are available on request from the corresponding author.

## Supplementary Materials

The following supporting information can be downloaded at: https://www.mdpi.com/article/10.3390/ijms24020922/s1.
