# Supplementary material for: Synaptic Activity Regulates Mitochondrial Iron Metabolism to Enhance Neuronal Bioenergetics
Source: Int J Mol Sci. 2023 Jan 4;24(2):922. doi: 10.3390/ijms24020922 (PMC9864932; doi:10.3390/ijms24020922)
Supplement: Supplementary file 1 [file ijms-24-00922-s001.zip › Fig S1.pdf]

Figure Supplementary 1

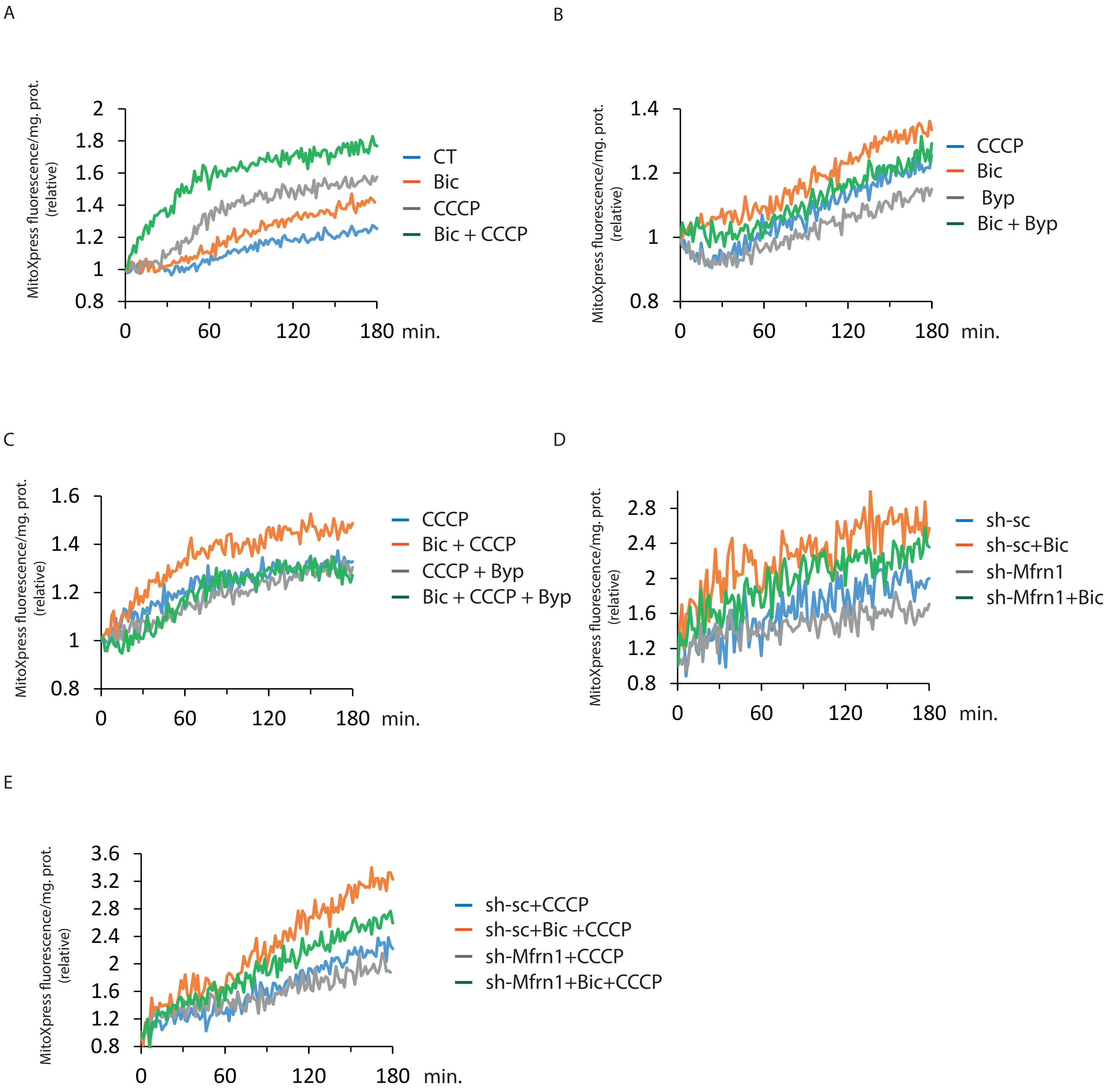

Fig. S1. Representative traces of MitoXPress Xtra fluorescence throug the time in neurons trated as indicated.
