# Supplementary material for: Synaptic Activity Regulates Mitochondrial Iron Metabolism to Enhance Neuronal Bioenergetics
Source: Int J Mol Sci. 2023 Jan 4;24(2):922. doi: 10.3390/ijms24020922 (PMC9864932; doi:10.3390/ijms24020922)
Supplement: Supplementary file 1 [file ijms-24-00922-s001.zip › Fig S2.pdf]

Figure Supplementary 2

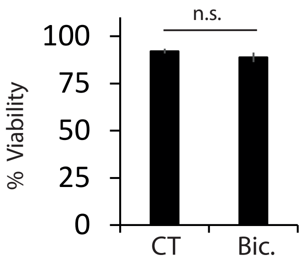

Fig. S2. Percentage of viability in neuronal cultures stimulated with Bic for 24 h or left unstimulated. Cell death was determined by staining nuclei with DAPI and counting pyknotic or fragmented nuclei. (n= 7 independent experiments). *p < 0.05, two-tailed Student's t-test.
