# Supplementary material for: Synaptic Activity Regulates Mitochondrial Iron Metabolism to Enhance Neuronal Bioenergetics
Source: Int J Mol Sci. 2023 Jan 4;24(2):922. doi: 10.3390/ijms24020922 (PMC9864932; doi:10.3390/ijms24020922)
Supplement: Supplementary file 1 [file ijms-24-00922-s001.zip › Fig S3.pdf]

Figure Supplementary 3

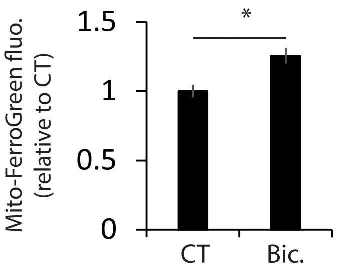

Fig. S3. Mitochondrial iron level in neurites. Mitochondrial ferrous content was analyzed in control and 24 h stimulated neurons (n= 4 independent experiments). *$p < 0.05$, two-tailed Student's t-test.
